# 转移抑制与metastamiR调节的联系

**DOI:** 10.3779/j.issn.1009-3419.2010.02.12

**Published:** 2010-02-20

**Authors:** Mick D. EDMONDS, Douglas R. HURST, Danny R. WELCH, 伟强 王, 书军 李

**Affiliations:** 1 Department of Pathology; University of Alabama at Birmingham; Birmingham, AL USA; 2 Cell Biology; University of Alabama at Birmingham; Birmingham, AL USA; 3 Pharmacology/Toxicology; University of Alabama at Birmingham; Birmingham, AL USA; 4 Comprehensive Cancer Center; University of Alabama at Birmingham; Birmingham, AL USA; 5 National Foundation for Cancer Research-Center for Metastasis Research; University of Alabama at Birmingham; Birmingham, AL USA; 6 天津医科大学总医院，天津市肺癌研究所，天津市肺癌转移与肿瘤微环境重点实验室

**Keywords:** 转移抑制, 小分子RNA, BRMS1, metastamiR

【缩写】 BRMS1：乳腺癌转移抑制因子1；HDAC：组蛋白去乙酰化酶；EGFR：表皮生长因子受体；miRNA：小分子RNA；miR：小分子RNA；pri-miRNA：初始小分子RNA；pre-miRNA：小分子RNA前体

癌症转移需要转移级联的各个步骤中多个基因的协调表达。调节这些遗传程序的分子可能在多个水平上影响转移。乳腺癌转移抑制因子1（BRMS1）通过调节许多蛋白质编码的、转移相关的基因以及转移调节小分子RNA（术语为metastamiR）来抑制级联中的多个步骤进而抑制转移。在此专题中，我们将重点阐述BRMS1的生物学特性与metastamiR调节之间的联系。

## 引言

癌症转移的形成需完成几个步骤，且每个步骤均为不同的且高度协调的遗传程序。转移中的每一步均是限速的，这意味着任何一步受阻，肿瘤细胞即不能进展至下一步。转移抑制因子是一组基因产物，根据定义，它们可阻断转移，但不能阻止原发肿瘤的形成，并有可能成为干预癌细胞最致命特性——转移的靶点^[1-4]^。BRMS1转移抑制因子主要通过改变SIN3：组蛋白去乙酰化酶（HDAC）染色质重构复合体来调节多个转移相关基因，进而抑制转移级联的多个步骤^[5-8]^。另外，BRMS1可协调调节多个metastamiR（转移相关小分子RNA），包括上调转移抑制miRNA以及下调转移促进miRNA^[9]^。

小分子RNA（miRNA）是一类非编码RNA基因^[10-12]^。miRNA主要在转录后水平上调节基因表达。miRNA基因主要由RNA聚合酶Ⅱ进行转录，转录时形成一个典型的发卡样结构（pri-miRNA）。RNAse Ⅲ Drosha连同共同因子DiGeorge综合征关键区域基因8（DGCR8），可以识别并剪切发卡结构，并生成pre-miRNA转运至细胞浆。Dicer酶和其它核糖核蛋白可进一步使发卡结构形成成熟的约22个核苷酸的miRNA，miRNA与RNA诱导沉默复合体（RISC）结合后，可靶向作用于mRNA的3'UTR，进而调节翻译^[13, 14]^。鉴于成熟的miRNA序列短，特定的miRNA理论上可靶向作用于数以百计的mRNA，因此miRNA为许多正常和病理细胞过程的关键调节因子。miRNA在哺乳动物细胞中被发现后不久，在癌症中也发现了miRNA的调节异常。在慢性淋巴细胞白血病中miR-15/16有频繁下调或缺失^[15]^。之后，有研究发现在大多数癌症中miRNA既有促癌作用，也有抑癌作用^[16]^。新近，研究发现数个miRNA在不依赖原发肿瘤发生的情况下即可调节癌转移过程^[17]^。

两篇新近论文促使作者撰成本篇短评。首先，BRMS1可上调转移抑制性miR-146a/b的表达，而miR- 146a/b单独即可抑制乳腺癌的转移^[18]^。其次，BRMS1可协同调节多个metastamiR的表达^[9]^。在这篇综述中，我们将回顾已知的BRMS1的metastamiR靶点以及BRMS1的这些功能如何与已知的BRMS1的生物学作用相一致。

## BRMS1调节转移相关基因

采用微细胞介导染色体转移技术将11号染色体转入转移性乳腺癌细胞可抑制转移，由此发现了BRMS1^[19]^。紧随此项研究，采用差异显示杂交技术鉴定出了差异表达基因^[20]^。利用这些分析，全长的BRMS1被克隆。当BRMS1转染至转移性细胞中时，原位乳腺癌、黑色素瘤和非小细胞肺癌仍能继续生长，但它们的转移能力下降^[20-24]^。

有关生物学机制，我们及其他研究者均发现BRMS1可改变转移的不同步骤所需的几种组分：恢复缝隙连接的细胞间信息交流^[25]^、促进失巢凋亡^[22]^、抑制在软琼脂中的生长^[20, 26]^及转移/侵袭^[22, 23, 26]^。在分子水平上，当BRMS1重新表达时，可改变转录组^[27]^和蛋白质组^[28, 29]^。这些变化包括（但不仅局限于此）：表皮生长因子受体（EGFR; ^[8, 30]^）、骨桥蛋白（OPN; ^[8, 31-33]^）、尿激酶型纤溶酶原激活剂（uPA; ^[34, 35]^）、C-X-C趋化因子受体4（CXCR4;^[36]^）和成束蛋白（fascin）^[37]^。研究认为这些改变主要是由于BRMS1与SIN3:HDAC染色质重构复合体之间的相互作用^[5-8, 38]^。总之，BRMS1转移抑制机制的假说认为BRMS1是转移相关基因表达的表观遗传学调节因子。

## MetastamiR—miRNA功能的新发现

我们猜想，BRMS1除了调控转移相关蛋白编码基因外，也可调控包括miRNA在内的非编码基因。利用miRNA杂交技术，研究发现BRMS1可调节miRNA的一个亚组，此亚组中的数个miRNA可被称为metastamiR。有趣的是，通常当促进转移的metastamiR减少时，抑制转移的metastamiR往往会增多^[9]^。

Ma, Weinberg及其同事首次发现并报道了一种metastamiR-miR-10b的存在^[39]^。miR-10b可促进离体与在体的肿瘤细胞侵袭，且不影响肿瘤细胞的生存或增殖。重要的是，促进了在体的转移（备注：离体的转移无法被测量；仅可应用体外实验来模拟参与转移的步骤）。其它促转移metastamiRs亦已被体内功能数据证实，包括：miR-21，-143，-182，-373和520c^[40]^。

miR-143和-182可分别促进肝细胞癌和黑色素瘤的转移^[41, 42]^。miR-143可通过NFκB上调其表达，并可降低细胞间粘附。miR-182可促进体内迁移，且是位于染色体7q31-q34上的部分microRNA簇，此外，microRNA簇还包括miR-96和miR-183（miR-183-96-182）。在晚期人黑色素瘤中，这一区域扩增频繁^[43]^，这进一步支持此microRNA簇在浸润行为中的作用。有趣的是，此microRNA簇与人进行性听力丧失有关^[44, 45]^。

对理解miR-182在控制癌细胞行为中作用的重要的补充是，miRNA基因常位于染色体的近端。miRNA簇在基因上相互连接，通过一个共同的启动子频繁转录，并可产生多顺反子初级转录产物^[46]^。因此，单个microRNA在协同网络中表达，这意味着单个microRNA与其它microRNA的共同表达使得分析microRNA簇中单个的microRNA的功能复杂化。因此，在癌症中，当对miR-182进行单独检测时，miR-96和-183可能也参与到其中。然而，据我们所知，目前尚无人采用功能分析的方法对任一microRNA簇的单体和/或联合功能进行系统的研究。我们认为这些分析对确定microRNA在癌症生物学中的作用至关重要。

miR-21可促进侵袭和迁移，同时减少凋亡。采用antagomir^[47]^短暂性敲除miR-21可显著降低乳腺癌和结直肠癌细胞向肺的实验性转移^[48]^。利用miRNA表达数据库，通过对转导有非转移性MCF7的人乳腺癌细胞进行高通量分析，鉴定出了miR-373和-520c。应用transwell迁移实验筛选转导子发现，miR-373和-520c是体外迁移和侵袭的促进因子^[49]^。实验性转移检测进一步显示miR-373和-520c均为真正的metastamiR，因为它们可促进肿瘤转移至多个部位。

另一类metastamiR可抑制转移。转移抑制metastamiR包括：miR-31^[50]^，-146a/b^[18]^，-206^[51]^和-335^[51]^。许多研究小组发现miR-146在炎症中起作用^[52-55]^。miR-146a和b均可抑制乳腺癌细胞侵袭和迁移，其机制可能是通过靶向作用于IRAK1和TRAF6而下调NFκB ^[53]^。miR-146a/b通过促进侵袭和转移的EGFR^[18]^和/或信号分子ROCK1^[56]^的表达，可进一步降低癌细胞的转移潜能。扩展至体内的这些研究显示miR-146a和b均可抑制乳腺癌细胞系中的实验性转移^[18]^。

miR-206和-335是首先被鉴定的抑制性metastamiR。Tavazoie等比较了源于人乳腺癌细胞系MDA-MB-231的各转移性变异体中miRNA的表达^[51]^。在转移性变异体中有6个特定miRNA的表达同时处于低水平。其中3个metastamiR——miR-335、-126和-206，可抑制转移；而miR-126也可抑制细胞增殖和肿瘤发生。因此，只有miR-335和-206可被归类为转移抑制因子。miR-335和-206抑制转移的关键步骤是抑制侵袭和迁移。

尽管大部分metastmiRs似乎在肿瘤细胞的侵袭和转移中发挥着关键作用，但迄今为止只有1个metastmiR被证明在转移级联的多个步骤中起作用。最近有报道称miR-31可抑制细胞侵袭、促进失巢凋亡并抑制异位定殖^[50]^，并使原位乳腺癌模型的肺转移减少95%，同时依然允许原位生长。通过基因个体发育分析发现，miR-31可抑制卷曲蛋白3（Fzd3）、整合素α-5（ITGA5）、肌球蛋白磷酸酶-Rho-相互作用蛋白（M-RIP）、基质金属蛋白酶16（MMP 16）、根蛋白（RDX）和RhoA。

我们认为，更多microRNA的表达与肿瘤的浸润、侵袭、转移和存活相关。然而，我们的讨论仅限于体内功能数据已发表且对转移具有选择性调节作用的microRNA。无疑，metastamiR的数量还会继续增加。

尽管每一metastamiR均存在多个靶点，但最终结果皆为转移的增多抑或是减少。有趣的是，已知的转录抑制因子BRMS1，既可增加转移抑制metastamiR的表达又可降低转移促进metastamiR的表达。BRMS1如何协同调节如此众多的转移相关的遗传过程，目前仍不明了。许多metastamiR可能通过数量有限的信号通路联系起来，而BRSM1通过直接和间接的机制来调节它们的表达。

## 在多个水平上调节的关键性转移信号通路

MetastamiR和转移抑制因子均可作用于转移级联的特定步骤。已有研究证实有11个miRNA可促进或阻断转移。这一数量有可能增加，因为已发现至少有20多个miRNA可影响转移的所有重要步骤，如上皮-间质转化（EMT）、细胞凋亡与血管生成。BRMS1是第一个被发现的可调节miRNA的转移抑制因子。同样，25个其它转移抑制因子中的一部分亦可能直接或间接影响metastamiR的表达。

更有意义的是BRMS1和miR-31间的相似性。两者均可阻断转移级联的多个步骤；两者均可阻断异位定殖；以及两者均可改变几种转移相关蛋白的表达和信号通路。

这些发现连同有关miRNA和转移相关级联反应的文献的大量出现，促使我们将这一综述的重点扩展至这一领域的发展现状。而且，几项临床研究已发现miRNA的表达与复发、转移的发展和/或生存之间存在相关性^[57]^。因此，我们的目的是关注转移调节小分子RNA（metastamirs）的证据、其存在的意义以及它们的发现过程中呈现的一些技术和理论的思索。

miR-10b最终通过激活RhoC^[39]^对转移起正性调节作用，RhoC在BRMS1细胞中的表达也下降^[9]^。BRMS1、miR-10b与RhoC之间的联系值得深思；然而，我们在阐述其间关系时仍需谨慎。上皮-间质转化诱导转录因子TWIST1，是miR-10b的正性调节因子。表达BRMS1的细胞可下调TWIST1和miR-10b的表达。由于TWIST1和miR- 10b启动子仍未完全被鉴定，目前无法确定BRMS1是仅作用于TWIST1还是作用于TWIST1和miR-10b。

同样，参与miR-10b级联的数个分子同时受BRMS1的调节。例如，EGFR通过Jak/stat信号（不依赖Erk/MEK信号T）可刺激TWIS进而引起EMT ^[58]^。BRMS1可直接（通过降低受体表达）和间接（通过减弱磷酸肌醇信号）下调EGF信号^[30]^。EGFR表达和TWIST-miR-10b-RhoC轴的降低可能是BRMS1调节miR-146a/b的结果，miR-146a和miR-146b均可靶向作用于EGFR^[18]^。

我们以及来自多个其它实验室的数据均清晰显示BRMS1对转移的调节是多层次的。在细胞内对基因的直接及间接作用都是可操控的。BRMS1可以在转录水平或通过一种转录中间体直接改变基因表达（如：通过改变转录因子的表达，像TWIST）。同时，BRMS1也可通过如上所述的调控转录或通过改变miRNA的表达而减少翻译以调节蛋白表达。

## 结论

对多细胞机体来说，最重要的是细胞不会迁移至其它部位并与其它组织整合。那样会造成混乱并破坏动态平衡。失效的安全冗余（fail safe redundancies）似乎可成为控制癌转移的通路。但是，这一冗余的出现需付出代价。尽管我们对癌转移研究所呈现的成果已初步形成复杂的转移调节系统，但我们的发现（连同许多其它实验室的发现）同时又提出了如下几个问题：

•为什么细胞可产生多个调节因子以操控单个分子？在不同的作用机制和分子之间存在什么类型的相互作用？

•为什么细胞可产生具有众多靶点的单个分子（像miRNA）？是否存在特异性的靶点？是否还有其它共同因子参与？miRNA表达的化学计量学是否重要？

•metastamiR是否会成为有用的临床治疗靶点？如果会，miRNA的混乱会造成更多还是更少的脱靶效应？

•是否所有的转移抑制因子均可改变metastamiR的表达？转移抑制因子“通路”中是否存在重叠现象？

•外源miRNA与内源miRNA是否有相似的作用？当某一miRNA存在于簇中时，人为再引入单个miRNA是否会产生生物学效应？或者是否应该对miRNA进行成对的（或更有序的）研究？

•在转移中pri-miRNA和pre-miRNA是否也起作用？

•miRNA（metastamiR）以及转移抑制因子和/或促进因子的反馈机制是什么？
